# Supporting healthy pregnancies: Examining variations in nutrition, weight management and substance abuse advice provision by prenatal care providers in Alberta, Canada. A study using the All Our Families cohort

**DOI:** 10.1371/journal.pone.0210290

**Published:** 2019-01-07

**Authors:** Shainur Premji, Sheila W. McDonald, Carol Zaychkowsky, Jennifer D. Zwicker

**Affiliations:** 1 The School of Public Policy, University of Calgary, Calgary, Alberta, Canada; 2 Cumming School of Medicine, University of Calgary, Calgary, Alberta, Canada; 3 Population, Public and Indigenous Health, Alberta Health Services, Calgary, Alberta, Canada; 4 Faculty of Kinesiology, University of Calgary, Calgary, Alberta, Canada; BITS Pilani, INDIA

## Abstract

**Background:**

Pregnancy is a critical time for fetal development, and education of women regarding healthy lifestyle choices is an important function for prenatal care providers, those that provide care to women during pregnancy. Within Canada, women choose to receive pregnancy care from one of a variety of publicly funded care providers. This study examines the association between the type of care provider(s) seen during pregnancy and the provision of advice related to nutrition, weight management and substance abuse.

**Methods:**

Using data from the Alberta-based All Our Families prospective pregnancy cohort, we conducted bivariate and multivariate analyses to determine the likelihood of receiving advice related to nutrition, weight management, and substance abuse across provider(s) seen.

**Results:**

Of 3341 women in our sample, 38% saw a single provider during pregnancy and 56% received care from multiple providers. Advice on nutrition was more likely to be provided across all providers, while weight management and substance abuse was less frequently and less consistently discussed. Relative to doctors in low-risk maternity clinics, midwives were most likely to provide nutrition (OR: 3.09, 95% CI: 1.19–8.01) and weight management (OR: 1.99, 95% CI: 1.13–3.50) advice to women.

**Conclusion:**

Findings suggest that the type of prenatal advice received by women depends on the provider(s) seen during pregnancy. Substance abuse was least likely to be discussed across providers, suggesting important implications given recent cannabis legalization.

## Introduction

The prenatal period, which takes place between conception and birth, is a critical time for fetal development, and healthy growth depends heavily on maternal behaviours during this phase. For this reason, pregnancy is promoted as a *teachable moment*, a time when women are effortlessly motivated to adopt healthier behaviours in order to influence positive outcomes for themselves and their offspring [1]. This provides an opportunity for care providers to educate women around the healthy lifestyle choices they can implement to support their and their offspring’s health and avoid preventable adversities [1–5]. The Society for Obstetricians and Gynaecologists in Canada (SOGC) encourages providers to have an open, communicative relationship with their patients, allowing them to ask questions and discuss issues to support informed decision making regarding their health during pregnancy [4]. The SOGC further encourages women to inform their care provider as soon as they suspect they are pregnant and to be seen within the first trimester [4].

Within Alberta, Canada, women have a choice in which publicly funded prenatal care provider they see [6]. The Alberta Prenatal Record is used to by care providers to record a woman's medical history and lifestyle factors that can impact their pregnancy and postpartum experience [7]. Recently, there has been a decline in family doctors providing prenatal care services and an expansion of publicly funded midwifery services across Canadian provinces [8, 9]. Family physicians are encouraged to refer women to low-risk maternity clinics, unless medically necessary for them to provide care themselves [10]. While having several choices can be considered beneficial for women, this can make prenatal care complex and confusing for both women and providers [11]. Referral pathways between providers can be unclear and information sharing can be fragmented [6], leading to a detriment in the quality of information and services offered to women. Furthermore, research indicates that different providers have varying styles and foci of services due to differing scopes of practice [6, 12–14]. This may contribute to inconsistencies in prenatal advice provided. For example, midwives tend to provide less medicalized care compared to physicians, and obstetricians tend to use more medical and surgical interventions compared to other providers [6, 12–14]. Despite the differences in service provision and foci, prenatal advice remains important for all women.

The aim of this study was to examine the association between the type of care provider(s) seen during pregnancy and the provision of prenatal advice to women. According to their most recent guidelines, the SOGC recommends that nutritional assessment and monitoring be part of every prenatal plan, and that providers discuss lifestyle factors, including nutrition, weight management and substance abuse during the initial prenatal visit [4]. Care providers are further reminded, within the Alberta Prenatal Record, to talk to women regarding their nutrition, weight management and substance use [7]. Consequently, this study placed a focus on whether women reported receiving advice related to these healthy behaviours from their prenatal care providers. The following analysis suggests that advice received by women varies depending on the care provider(s) seen during pregnancy.

## Methods

A cohort analysis using data from the Alberta-based All Our Families (AOF) prospective pregnancy cohort (n = 3300) was conducted. AOF was designed to gather information for the prenatal period through to early childhood and includes outcomes for both mothers and their children. Full details regarding recruitment, data collection and analysis can be found elsewhere [15, 16]. Briefly: women less than 25 weeks’ gestation were recruited between 2008 and 2011 through primary health clinics, laboratory services, and via poster displays. Women were asked to complete questionnaires twice during pregnancy and at multiple time points following childbirth up to 5 years postpartum [15, 16]. Data collection is ongoing for the 8-year follow-up period. Between 32 and 36 weeks’ gestation, women were asked to report the type of healthcare provider(s) they saw during pregnancy. This identified whether they received prenatal care from a single or multiple providers. Providers included family doctors, doctors in low-risk maternity clinics (DLRMC), obstetricians, midwives, and walk-in clinic doctors. Women also reported whether or not they received advice related to healthy pregnancy behaviours, constituting outcome variables for this study, including: nutrition or vitamins and minerals (‘nutrition’); exercise or weight gain (‘weight management’), and smoking, drinking, or drugs prevention (‘substance abuse’).

Descriptive analysis took place using frequencies, percentages and 95% confidence intervals (CI). Bivariate analyses and Chi-square tests of association examined the relationship between various maternal and pregnancy characteristics and whether prenatal care was received from a single versus multiple provider(s). To determine the likelihood of receiving versus not receiving nutrition, weight management and substance abuse advice, crude odds and 95% CI were calculated by provider(s) seen. Adjusted odds ratios (ORs) using multivariable logistic regression models compared the odds of receiving advice by provider(s) versus a DLRMC. All models were adjusted for the following covariates based on consultation with content experts: prenatal class attendance, number of prenatal visits, initial prenatal visit taking place during the first trimester, parity, ethnicity, time in Canada, visit with a nutritionist/ dietitian (nutrition model only), and history of substance abuse problems (substance abuse model only). A manual backwards stepwise approach was taken to arrive at final parsimonious models. We chose the DLRMC as our reference group because it was the most commonly seen provider in our sample. Information for these covariates were derived from the self-reported AOF questionnaires. For each model, we additionally examined whether parity, assisted reproduction status, maternal age or pre-pregnancy BMI (nutrition and weight management models only) modified the relationship between provider(s) seen and type of advice received. Due to low sample sizes, pre-pregnancy BMI categories were combined as follows: under/ normal weight and overweight/ obese.

Ethics approval for this study was received by the Conjoint Faculties Research Ethics Board at the University of Calgary. Data cleaning and analysis took place using Stata 14 within the Secondary Analysis to Generate Evidence (SAGE) virtual environment, administered through PolicyWise for Children and Families.

## Results

Of the 3341 women in the sample, 1283 (38%) saw a single provider during pregnancy and 1859 (56%) received care from multiple providers (Table 1). Women were, on average, 31 years of age at delivery, and 97% were partnered. The majority of the sample was educated, with at least a high school-level diploma or higher (88%), were of Caucasian descent (78%), were born in or had lived in Canada at least five years (89%) and had an annual gross household income of $60,000 or greater. Women that were less educated (p = 0.014), with lower annual income (p = 0.001) and who were multiparous (p = 0.027) were more likely to receive care from a single, versus multiple, provider(s) during pregnancy.

**Table 1:** Sample characteristics.

	Whole Population (n = 3341)	Single Provider (n = 1283)	Multiple Providers (n = 1859)	p-value^a^
	**Mean (SD)**			
**Maternal age**	31.2 (4.4)	31.2 (4.4)	31.3 (4.4)	0.587
	**n (%)**			
**Marital status**				
Partnered	3245 (97.1%)	1240 (97.4%)	1808 (97.5%)	0.668
Not-Partnered	62 (1.9%)	21 (1.6%)	27 (1.5%)	
**Education**				
≤High School	363 (10.9%)	149 (11.7%)	167 (9.0%)	0.014
>High School	2946 (88.2%)	1111 (87.5%)	1669 (90.0%)	
**Ethnicity**				
White/Caucasian	2604 (77.9%)	993 (78.2%)	1469 (79.2%)	0.416
Other	703 (21.0%)	267 (21.0%)	367 (19.8%)	
**Time in Canada**				
Born in Canada/ lived ≥5 years	2979 (89.2%)	1128 (88.8%)	1673 (90.2%)	0.221
Lived <5 years	318 (9.5%)	125 (9.8%)	159 (8.6%)	
**Annual household income**				
<$60,000	585 (17.5%)	240 (18.9%)	269 (14.5%)	0.001
≥$60,000	2625 (78.6%)	987 (77.7%)	1508 (81.3%)	
**Parity**				
Primaparous	1609 (48.2%)	581 (45.8%)	923 (49.8%)	0.027
Multiparous	1686 (50.5%)	672 (52.9%)	908 (49.0%)	

^a^Differences examined using chi-square tests of association

### Nutrition

**Fig 1:**
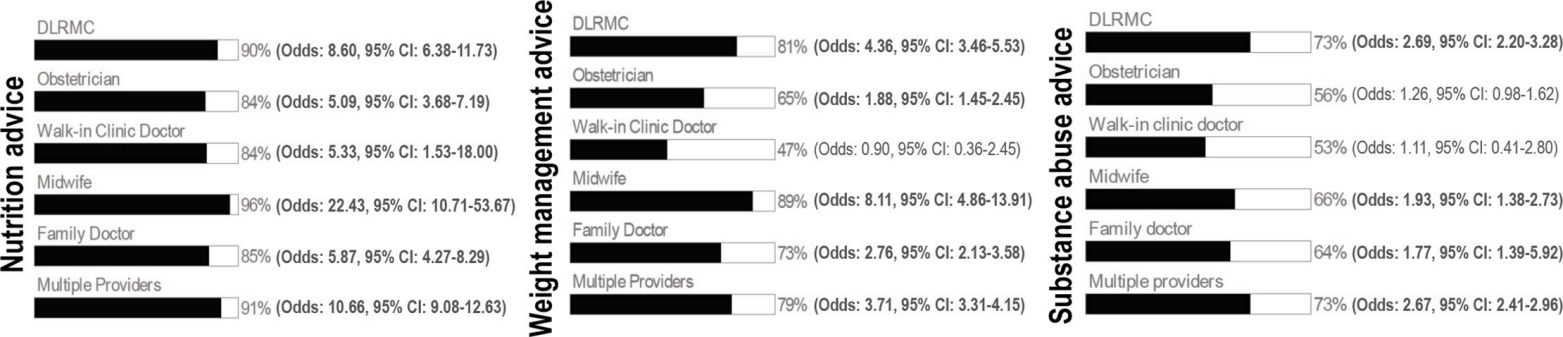
Proportion, odds and 95% CI of women reported to receive, verses not receive, advice from a prenatal care provider.

Between 84% and 96% of women reported receiving advice related to nutrition by their care provider(s) during pregnancy (Fig 1). Midwives were most likely to provide than not provide advice related to nutrition (odds: 22.43, 95% CI: 10.71–53.67), and three times more likely to provide advice related to nutrition than DLRMCs (OR: 3.09, 95% CI: 1.19–8.01; Table 2). Compared to DLRMCs, obstetricians had 48% lower odds of providing nutrition advice to women (OR: 0.52, 95% CI: 0.32–0.85) and family doctors had 64% (OR: 0.36, 95% CI: 0.19–0.67) and 54% (OR: 0.46, 95% CI: 0.27–0.80) lower odds of providing nutrition advice to multiparous women and women categorized as under or normal weight pre-pregnancy, respectively.

**Table 2:** Final multivariable logistic regression results.

	Unadjusted OR (95% CI)	Adjusted OR^a^ (95% CI)	
**Nutrition advice**			
DLRMC	1.00	1.00	
Obstetrician	0.59 (0.38–0.91)	0.52 (0.32–0.85)	
Walk-in Clinic Doctor	0.62 (0.17–2.20)	0.62 (0.13–2.90)	
Midwife–Women with no assisted reproduction	2.95 (1.24–7.02)	3.09 (1.19–8.01)	
Midwife–Women with assisted reproduction	0.50 (0.03–9.08)	0.66 (0.03–12.38)	
Family doctor–Multiparous women	0.42 (0.24–0.75)	0.36 (0.19–0.67)	
Family doctor–Primaparous women	1.51 (0.71–3.17)	1.25 (0.54–2.90)	
Family doctor–Women classified as underweight/ normal pre-pregnancy BMI	0.57 (0.35–0.95)	0.46 (0.27–0.80)	
Family doctor–Women classified as overweight/ obese pre-pregnancy BMI	1.52 (0.61–3.76)	1.57 (0.54–4.54)	
Multiple Providers	1.24 (0.89–1.72)	1.04 (0.72–1.51)	
**Weight management advice**			
DLRMC	1.00		1.00
Obstetrician	0.43 (0.31–0.60)		0.46 (0.32–0.67)
Walk-in Clinic Doctor	0.21 (0.08–0.52)		0.24 (0.08–0.72)
Midwife	1.86 (1.08–3.19)		1.99 (1.13–3.50)
Family Doctor	0.63 (0.45–0.89)		0.72 (0.49–1.05)
Multiple Providers	0.85 (0.66–1.09)		0.82 (0.62–1.07)
**Substance abuse advice**			
DLRMC	1.00		1.00
Obstetrician	0.47 (0.34–0.64)		0.48 (0.35–0.66)
Walk-in Clinic Doctor	0.41 (0.16–1.04)		0.41 (0.15–1.11)
Midwife	0.72 (0.49–1.05)		0.82 (0.56–1.20)
Family Doctor	0.66 (0.49–0.89)		0.69 (0.51–0.95)
Multiple Providers	0.99 (0.80–1.24)		1.00 (0.81–1.26)

^a^Covariates: prenatal class attendance, number of prenatal visits, initial prenatal visit taking place during the first trimester, parity, ethnicity, time in Canada, visit with a nutritionist/ dietitian (nutrition model only), and history of substance abuse problems (substance abuse model only)

### Weight management

Between 47% and 89% of women reported receiving weight management advice from their prenatal care provider(s) during pregnancy (Fig 1). Midwives were most likely to provide than not provide advice related to weight management (odds: 8.11, 95% CI: 4.86–13.91); whereas walk-in clinic doctors were equally likely to provide, as not provide, advice related to weight management (odds: 0.90, 95% CI: 0.36–2.45). Compared to DLRMCs, obstetricians had 54% lower odds of providing weight management advice to women (OR: 0.46, 95% CI: 0.32–0.67) during pregnancy (Table 2), and midwives had twice the odds of providing weight management advice to women (OR: 1.99, 95% CI: 1.13–3.50). There was no evidence that parity, assisted reproduction status, pre-pregnancy BMI or maternal age modified the relationship between provider(s) seen during pregnancy and weight management advice received by women.

### Substance abuse

Between 53% and 73% of women reported receiving advice related to substance abuse during pregnancy (Fig 1). Women reported that DLRMCs were most likely to provide, than not provide, substance abuse advice during pregnancy (odds: 2.69, 95% CI: 2.20–3.28), whereas obstetricians (odds: 1.26, 95% CI: 0.98–1.62) and walk-in clinic doctors (odds: 1.11, 95% CI: 0.41–2.80) were reported equally likely to discuss, as not discuss, substance abuse with women. Relative to DLRMCs, obstetricians had 52% lower odds (OR: 0.48, 95% CI: 0.35–0.66), and family doctors had 31% lower odds (OR: 0.69, 95% CI: 0.51–0.95), of discussing substance abuse with women during pregnancy (Table 2). There was no evidence that parity, assisted reproduction status, or maternal age modified the relationship between provider(s) seen during pregnancy and substance abuse advice received by women.

## Discussion

The aim of this study was to examine the association between the type of care provider(s) seen during pregnancy and the provision of prenatal advice to women. Women have the ability to receive care from publicly funded prenatal care providers with different foci and scopes of practice [6, 12–14]. This study suggests that the type of prenatal advice received by women was influenced by the provider(s) they saw during pregnancy. Since women were able to see multiple providers during their pregnancy, we expected that seeing multiple providers would increase their likelihood of receiving advice. However, within our sample the likelihood of receiving advice among women who saw multiple providers was not higher than the likelihood of receiving advice if women received care from a DLRMC. This suggests that seeing more providers does not necessarily mean receiving more complete information. Furthermore, previous studies have shown that multiple providers may share conflicting messages with women, resulting in increased confusion and anxiety [11]. Providing inconsistent information and duplication of care may also lead to increased costs for the health care system and should be avoided, as much as possible [6].

Overall, women consistently reported receiving advice related to nutrition (84%-96%), regardless of the provider(s) they saw for their prenatal care. However, a lower and more varied proportion of women (47%-89%) reported discussing weight management with their provider(s) during pregnancy, whereas substance abuse prevention appeared to be least consistently discussed (53%-73%). Of all providers, midwives were most likely to provide advice related to nutrition to women. This may be a result of longer visits (i.e., approximately 30–45 minutes per visit, compared to 15 minutes for family physicians and 10 minutes for obstetricians [17]). Given the SOGC encourages nutritional assessments for all women [4], the consistency reported by women in receiving this type of advice, regardless of the length of visit with their provider(s), is an encouraging finding.

In contrast to 90% of women that reported discussing nutrition, only 81% reported discussing weight management with their DLRMC. Findings on weight management indicate that obstetricians and walk-in doctors were significantly less likely than DLRMC to discuss this issue with women. These results may reflect the nature of obstetric and walk-in clinic care and the specialized and acute nature of scopes of practice for these professions. Family physicians and multiple providers were equally likely as DLRMCs to discuss this type of advice. Similar to providing advice related to nutrition, midwives were reported as *most likely* to discuss weight management during pregnancy. While this could, again, be the result of longer visits, training may be a factor. Notably, the literature shows inconsistency in this finding across jurisdictions [18–20], suggesting that midwifery training plays a large part in preparing midwives to discuss issues related to obesity and weight management with women during pregnancy [18, 19, 21]. Our findings suggest a strong contribution from midwives in sharing weight management information in Alberta.

Exclusive of women that received care from midwives during pregnancy, only 47% to 81% of women reported discussing weight management with their provider(s). Our results were slightly higher than the McDonald [17] study, which stated between 39% and 64% of women discussed weight gain with their provider during pregnancy. Yet, this finding is concerning, given that many women still tend to gain more weight than recommended during pregnancy, thereby posing lifelong challenges for both themselves and their unborn child [22–24]. Other studies have established an association between pre-pregnancy BMI category and gestational weight gain [24, 25], and existing guidelines provide recommendations around gestational weight gain categories for women based on their pre-pregnancy BMI [25]. However, studies still confirm that the counselling provided to women is not consistent with gestational weight gain guidelines [17, 20]. McDonald [17] found that only 33% to 57% of women reported receiving correct weight gain advice according to guidelines, depending on prenatal care provider seen. This suggests more research is required to examine the content of messaging delivered by care providers in Alberta. Perhaps more education is also required with prenatal care providers to identify and overcome barriers associated with counseling women on gestational weight gain, such as a lack of comfort around how to raise this potentially sensitive topic [20].

With the exception of walk-in clinic doctors and obstetricians, women reported their provider(s) were more likely to provide, than not provide, advice related to substance abuse during pregnancy. Of the single providers seen during pregnancy, women reported that DLRMC were most likely to discuss than not discuss substance abuse prevention. Unfortunately, our findings show that both obstetricians and family doctors were statistically significantly less likely to discuss this issue relative to the DLRMC. These results align with other findings, where providers reported several barriers in discussing substance abuse with patients, including gaps in appropriate knowledge of up-to-date research in the area, such as safe cessation treatment options for expectant women; inconsistent organizational policies around how to address this issue; time constraints during appointments; and a fear of losing patient trust, or alienating disadvantaged women, by introducing this issue [18, 26].

While the prevalence of women reporting cigarette smoking during pregnancy has remained stable over time, reported cannabis use among pregnant women is trending upwards [27, 28]. Cannabis is the most commonly used illicit drug during pregnancy [27, 29]; researchers have found that cannabis use during pregnancy is relatively high, particularly among younger women and cigarette smokers [28, 30]. Approximately 5% of pregnant women reported illicit drug use during pregnancy in Canada [31], but other estimates suggest rates of cannabis use during pregnancy could range from 10–30% in various subsets of the population [32–34]. Given the recent legalization of cannabis in Canada, this presents a significant concern, in that there is currently low public awareness around the risks of cannabis intake during pregnancy and the associated harms [27, 35, 36]. This is an area for further research and policy improvement.

### Strengths and limitations

A major strength of this study is the ability to discern advice received by prenatal care provider(s) seen during pregnancy. Results from this study can be used to shape and refine prenatal care practices in Alberta. While this study contributes unique findings to the literature, several limitations do exist. It is possible that women reported seeing multiple providers because they were referred to an obstetrician or low-risk maternity clinic mid-pregnancy by their family doctor. In this situation, women may have received advice from one provider and not both; however, due to the context around how women were asked to report this information, it was not possible to attribute this advice to a single provider. This would lead to an underestimate in the effects found for single providers and an overestimate of effects found for multiple providers.

Another limitation is the potential for selection bias. In assessing the sociodemographic characteristics of women that did not provide any information around their prenatal care provider(s), we found that younger, non-partnered, non-Caucasian, low income women were less likely to answer which provider(s), if any, they saw during pregnancy (p<0.05). This also leads to an underestimation of the effects found in our study. Existing literature suggests women may face a fear of punishment and stigma in reporting their substance use to a healthcare provider [37, 38], further suggesting potential underestimates in effects found in this study. Additionally, advice received was participant reported and it is possible that advice provided was not retained by the participant. The provider perspective was unaccounted for in this study. Inclusion of this perspective in future studies can lead to enriched context around the barriers and enablers to discussing healthy lifestyle factors with pregnant women.

## Conclusion

This study suggests that the type of prenatal advice received by women was influenced by the provider(s) they saw during pregnancy. While family physicians have traditionally played a key role in education and behavior change for women, in recent years women have had access to information from various providers, raising the need for consistency in advice provision across all sources. Future work should focus on aligning and evaluating policy and practice guidelines in Alberta to improve standard communication of prenatal information and potentially reduce disparities in preventive health outcomes for mothers and children.
